# The effect of psychopathy on cooperative strategies in an iterated Prisoner’s Dilemma experiment with emotional feedback

**DOI:** 10.1038/s41598-019-38796-0

**Published:** 2019-02-19

**Authors:** Martina Testori, Thehela O. A. Harris, Rebecca B. Hoyle, Hedwig Eisenbarth

**Affiliations:** 1 University of Southampton, School of Mathematical Sciences, Southampton, SO17 1BJ UK; 2 University of Southampton, Faculty of Medicine, Southampton, SO17 1BJ UK; 3 University of Southampton, School of Psychology, Southampton, SO17 1BJ UK; 4 Victoria University of Wellington, School of Psychology, Wellington, 6011 New Zealand

## Abstract

As decision-making research becomes more popular, the inclusion of personality traits has emerged as a focal point for an exhaustive analysis of human behaviour. In this study, we investigate the impact of psychopathic traits on cooperation in an iterated Prisoner’s Dilemma game with emotional facial feedback. Firstly, we observed how receiving a facial feedback after each decision affected players with different psychopathic trait scores, and how being informed about the opponent’s identity influenced cooperative behaviour. Secondly, we analysed the strategies adopted by each player, and how these choices were correlated with their psychopathic traits. Although our results showed no effect of different emotional content in the feedback on cooperation, we observed more cooperative behaviours in those players who were told their opponent was another fellow human, compared to those who were told it was a computer. Moreover, fearless dominance had a very small but consistent negative effect on overall cooperation and on the tendency to maintain cooperative behaviours. We also found that players’ personality scores affected the strategies they chose to play throughout the game. Hence, our experiment adds complexity to the body of work investigating psychopathic traits and social interactions, considering not only the environment of facial feedback but also the role of deception in experimental games.

## Introduction

Significant dysfunction in interpersonal relations is a hallmark of psychopathy. It includes traits of callousness, guiltlessness, dishonesty and egocentricity^1^. These self-focused characteristics lend themselves to the significant behavioural differences seen in those high in psychopathic traits, especially in social situations.

The Prisoner’s Dilemma game (PD), and its variations, is a commonly used framework for researching personality traits within a social context. Several key studies have employed this game to investigate how psychopathic traits influence cooperation and defection. Interestingly, results have not all been consistent. One of the first studies to use the PD to investigate psychopathic traits surprisingly found that males high in primary and secondary psychopathic traits were not more likely to defect than those low in psychopathy^2^. A later study, however, found a significant negative correlation between psychopathic traits and cooperation, only in male participants^3^. Male prison samples showed a decreased cooperativeness among those high in psychopathy^4^, and high levels of impulsivity were also found to be strongly predictive of defective behaviours in the general population^5^. Specifically, two characteristics of psychopathy correlated negatively with cooperation as determined by the Psychopathic Personality Inventory Revised (PPI-R)^6^: Impulsive Nonconformity and Machiavellian Egocentricity. High levels of impulsivity were predictive of repeated defection, while machiavellianism was linked to higher overall defection. At the same time, Narcissistic personality traits have been found to differentially impact cooperative behaviour and overall outcome^7^.

An important aspect to consider in social interactions is individuals’ emotional response, especially in relation with personality traits. However, previous research on cooperation in the context of emotional feedback has not considered personality features. In psychopathy, emotional dysfunction has long been a defining characteristic^1^, and while there is some debate as to whether there is any dysfunction of emotional recognition^8^, the literature is fairly conclusive on a dysfunction in emotional reaction^9,10^. What remains to be seen is whether this emotional dysfunction affects cooperation in social interaction. One key study attempted to investigate a possible interaction of affective feedback and psychopathic traits in an iterated PD^11^. Participants performed two versions of the game, one with and one without verbal affective feedback. Results showed that high levels of psychopathy were significantly associated with reduced cooperation in the affective feedback version of the game, and positively associated with “CD” outcomes in both games (CD = player cooperates while the opponent defects).

Our study aims to build on these findings by implementing an experimental design which includes two different facial feedbacks (smiling and frowning faces) in four separate combinations (see Table 1). Given the evidence of the use of facial expression in determining partners’ trustworthiness^12^, and the association of happy faces with cooperation and sad faces with defection^13^, we hypothesise participants to be more likely to cooperate in the presence of happy facial feedback. As such, our first hypothesis is the following: highly psychopathic players will be less cooperative overall, especially in the presence of happy facial feedback, compared to low psychopathic participants.

In addition, we aim to investigate the effect of the opponent’s identity on cooperative behaviours. We implemented two game versions, a deception and a non-deception one, to observe differences in participants’ behaviour when the opponent is a fellow human or a computer. Playing versus a human opponent has been proven to elicit higher engagement and more positive emotional responses, when compared to computer opponents^14,15^. Thus, our second hypothesis states that: deceived players will show more cooperative behaviour compared to non-deceived ones.

Lastly, we are interested in estimating the strategies played throughout the game. A pioneer of IPD strategy analysis is Axelrod^16^. His main interest was to discover “how to play the game (IPD) well”. For this purpose, he invited professional game theorists from diverse disciplines to send him strategies that they considered successful. These strategies were then entered in a computer tournament in which each of them played against all the others for 200 rounds. As a result, he obtained a ranking of the most successful strategies devised up to that moment. Since then, strategies have been investigated in depth, however mainly to assess which was the most successful one. In our paper, on the other hand, we aim to identify which are the most used strategies, without focussing on how successful they are. Previous works focused on identifying players’ strategies through different techniques. Wedekind and Milinski^17^ were amongst the first to observed subjects’ strategies in both a simultaneous and an alternating Prisoner’s Dilemma game. Thereafter, various estimation techniques have been used to assess which strategy each subject was playing in different games, analysing players’ decisions directly^18–21^. In this experiment, we implemented the technique first presented by Dal Bó and Fréchette^18^ and we employed the same set of strategies considered in Fundenberg, Rand and Dreber^22^. We examined players’ strategies by comparing each player’s decisions throughout the game with some of the best known strategies in game theory^16^. We then analysed whether psychopathy scores correlate with the strategies adopted by the players. Under the assumption that highly psychopathic individuals are less prone to cooperate compared to low psychopathic individuals, we suppose that as psychopathic measures increase, the percentage of participants using defective strategies will increase. Therefore, our third hypothesis claims that high psychopathic individuals will adopt less cooperative strategies, compared to low psychopathic players.

## Experimental Design

Ethical approval was obtained from the Faculty of Medicine Ethics Committee and Research Governance Office at the University of Southampton, all experimental and survey procedures followed ethical guidelines from the declaration of Helsinki as well as guidelines of the institutional review board. All participants gave informed consent for participating in an online game. The experiment was constructed with four different conditions and two game versions. Firstly, we divided the complete sample equally into two groups: the Deceived and the Non-Deceived subgroups. Although in reality all participants played against a computer, deceived participants were told they were playing against a human opponent. Participants in the non-deception version were informed they were playing against a computer, however they were instructed to consider the stimuli they were going to see during the game (emotional feedback) as if they were received from real human opponents.

Secondly, we implemented four conditions symmetrically on the two subgroups divided according to the emotional content (happy, neutral and sad) of the facial feedback participants received after each round (Table 1). Videos for the facial feedbacks were taken from the Denver Intensity and Spontaneous Facial Action (DISFA) Database^23^. Two individuals were selected (one female, one male) and 10 different 300 ms snips were created for each of the female and the male actors and for each of the happy, neutral and sad expressions. Participants in each group, deceived and non-deceived, were randomly assigned to one of the four conditions. The game was programmed to give equal numbers of male and female opponents and equal numbers of participants in each of the four conditions.

**Table 1:** Emotional facial feedback in the four between-subjects conditions.

Player’s decision	Opponent’s emotional facial feedback			
	Condition 1	Condition 2	Condition 3	Condition 4
Cooperation	neutral	neutral	happy	happy
Defection	neutral	sad	neutral	sad

Participants were instructed to imagine a scenario in which they were the owner of an electronics shop, competing against another electronics store. In each of the 30 rounds, they were asked to assign to individual products either standard price (cooperation) or sale price (defection), knowing that their opponent was asked the same question. According to the decisions made by the player and the computer, four outcomes were possible (Table 2).

**Table 2:** Payoff matrix showing the percentage profit earned according to both players’ pricing decisions.

		Participant	
		Standard Price	Sale Price
Opponent	Standard Price	(30%, 30%)	(10%, 40%)
	Sale Price	(40%, 10%)	(20%, 20%)

The computer was programmed to play a tit-for-two-tats strategy, meaning that it cooperates until the opponent defects twice in a row, then it defects until the opponent cooperates again. In the last two rounds however, the computer was programmed to defect, as an attempt to simulate human behaviour. A page with the two decisions made and the consequent payoff was shown after each round. In addition to the payoff, participants were able to see a short video representing the presumed opponent’s face.

## Results

### Statistical Analysis

Three main questions were explored:

1. which factors affected the overall rate of cooperation during the game?
2. what persuaded players to continue cooperating?
3. can we approximate players’ behaviour with well known strategies?

To investigate the first two points, analyses were implemented on two different dependent variables: the overall percentage of cooperation for each participant over the 30 trials, and the number of times a participant cooperated immediately following a previous cooperation (“cooperation after cooperation” - CaC). The number of cooperations immediately following a defection was also regressed without reporting any significant effect (marginal effect of fearless dominance at the 0.1 level of significance, see online SI). The same analyses were implemented on the two dependent variables in parallel. The two explained variables were regressed against a fixed set of explanatory variables (see descriptive statistics in Table 3) which included *gender* (1 = female, 2 = male), as it has been found to be correlated with psychopathic traits, the four personality factors (*fearless dominance, self-centred impulsivity, coldheartedness* as dimensional scores, and *narcissism*), and the *maximise* variable describing, on a scale from 1 to 5, how much the players tried to maximise their own profit. To account for the effect of the two game versions, we included as fixed effect the *game version* variable (1 = deception, 2 = non-deception) which controlled the effect of the deception/non-deception games participants played. For the four conditions implemented in the game, we modelled them as a 2 × 2 factorial design, as explained in Table 4. Descriptive statistics of the variables are presented in Table 3, and the correlation coefficients can be found in Table 5.

**Table 3:** Descriptive statistics for the participant sample.

Variables	Min	Mean	Standard Deviation	Max	Cronbach’s *α*
gender	1	1.58	0.49	2	
maximise	1	4.26	0.90	5	
fearless dominance	17	34.67	7.51	56	0.83
self-centred impulsivity	16	30.91	5.89	49	0.73
coldheartedness	5	10.78	2.97	20	0.75
narcissism	0	0.08	0.09	0.33	0.54

**Table 4:** Two-by-two factorial analysis of the four conditions implemented.

Positive feedback	Negative feedback	
	Absence of negative feedback (0)	Presence of negative feedback (1)
absence of positive feedback (0)	condition 1	condition 2
presence of positive feedback (1)	condition 3	condition 4

**Table 5:** Bivariate correlation matrix among variables.

	Cooperation	CaC	Sum psychopathic traits	Self-centred impulsivity	Fearless dominance	Coldheartedness	Narcissism	Maximise
cooperation	1	—	—	—	—	—	—	—
CaC	0.90***	1	—	—	—	—	—	—
sum psychopathic traits	−0.15*	−0.12	1	—	—	—	—	—
self-centred impulsivity	−0.02	0.00	0.59***	1	—	—	—	—
fearless dominance	−0.17*	−0.18*	0.71***	−0.07	1	—	—	—
coldheartedness	−0.14.	−0.06	0.48***	0.28***	0.13.	1	—	—
narcissism	−0.03	−0.04	0.47***	0.25***	0.40***	0.27***	1	—
maximise	−0.17*	−0.15*	0.03	−0.01	0.01	0.18*	−0.01	1

Significance level: ‘***’ < 0.001 ‘**’ < 0.01 ‘*’ < 0.05 ‘.’ < 0.1; *sum psychopathic traits* is an aggregate measure of the PPI questionnaire.

The regression analysis consisted of two phases: model selection and model interpretation. Three models were considered, according to the structure of the dependent variables. Since both variables were produced from a series of zeros and ones, we considered a Generalised Linear Model (GLM), a Logistic (LM) and a Beta-Binomial (BBM) regression models as candidate and compared their fits to data. To select the best fitting model for our data, a repeated k-fold cross-validation was implemented. Finally, the best fitting model was implemented on the data and the results were interpreted.

A different approach was used for the analysis of the strategies adopted. In this case we followed the technique presented in Dal Bó and Fréchette^18^. Detail of the technique can be found in the online SI. In order to be able to infer the players’ strategies, we focused our attention on a sub-set of the multitude of existing games strategies (see more details in the online SI). Amongst them, we then repeated the analysis for those strategies which were most chosen and had a stronger correlation with the participants’ personality traits (Table 6).

**Table 6:** Description of the main six strategies considered.

Strategy	Abbreviation	Description
Tit for three tat	TF3T	Cooperate until the opponent defects three times in a row, then defect till the opponent cooperates again
Two tit for two tat	2TF2T	Cooperate until the opponent defects twice in a row, then defect till the opponent cooperates twice in a row
Grim	Grim	Cooperate until the opponent defects, then defect forever
Always defect	ALLD	Defect at each round

Interestingly, these strategies capture the most important aspects of IPD strategies^16^: punishment (Grim, TF3T), forgiveness and niceness (TF3T, 2TF2T). At the same time, ALLD represents a purely defective strategy, which is known to be the optimal strategy in an IPD and one of the most used one. The complete results for this pre-analysis can be found in the online SI. All data and code can be found on the osf platform.

### Cooperation analysis

The GLM was selected as the best model for both dependent variables using 5-fold cross-validation (see online SI).

To address the partialling issue^24^, we first regressed the dependent variables over the four personality trait variables (Table 7). The findings from this initial basic analysis were corroborated by the complete regressions subsequently conducted. Fearless dominance emerged as the only consistently statistically significant predictor: the lower people’s score in this factor of psychopathy, the more they cooperated over the 30 rounds and the more they persisted in cooperative strategies. As we hypothesised at the beginning, players who scored high in this psychopathy measure tended to cooperate less than low-psychopathic individuals. However, the analysis also shows that the different feedback conditions did not affect the rate of cooperation: receiving different types of emotional feedback did not affect individuals’ strategy, neither for the overall cooperation nor for CaC.

**Table 7:** GLM coefficients for participants’ overall cooperation and cooperation after cooperation.

DV:	Overall cooperation	CaC	Overall cooperation	CaC
intercept	0.42***	0.412***	0.66***	0.71***
	(0.02)	(0.02)	(0.12)	(0.13)
gender			−0.08.	−0.12*
			(0.04)	(0.05)
maximise			−0.05.	−0.05.
			(0.02)	(0.03)
fearless dominance	−0.01*	−0.01*	−0.01**	−0.01**
	(−003)	(0.00)	(0.00)	(0.00)
self-centred impulsivity	−0.00	−0.00	−0.00	−0.00
	(0.00)	(0.00)	(0.00)	(0.00)
coldheartedness	−0.01.	−0.01	−0.01	−0.00
	(0.01)	(0.01)	(0.01)	(0.01)
narcissism	0.28	0.23	0.21	0.16
	(0.28)	(0.33)	(0.27)	(0.32)
positive feedback			0.05	0.07
			(0.06)	(0.07)
negative feedback			0.04	0.09
			(0.07)	(0.07)
positive *negative feedback			−0.03	−0.10
			(0.09)	(0.10)
game version			−0.07.	−0.14**
			(0.04)	(0.05)

Standard errors for coefficients shown in parenthesis.

Significance level: ‘***’ < 0.001 ‘**’ < 0.01 ‘*’ < 0.05 and ‘.’ < 0.1.

To investigate whether participants showing different levels of psychopathy were differentially influenced by the facial feedbacks, we included the interaction terms between the cumulative score of psychopathy and the four conditions (Table 8). Results show a very small and marginally significant effect of two interaction terms (positive * negative * psychopathy and positive * psychopathy). Due to the small effect size and the marginal level of statistical significance (<0.1), the terms do not add any crucial contribution to the results previously found in Table 7. Hence, although we found that psychopathic traits do affect cooperation, we did not find any correlation with the facial feedback they were receiving during the game.

**Table 8:** Interaction terms in GLM models for participants’ overall cooperation and cooperation after cooperation, considering participants’ cumulative measure of psychopathy.

DV:	overall cooperation	cooperation after cooperation (CaC)
game version*sum psychopathic measures	0.00	0.00
	(0.00)	(0.00)
positive*negative feedback*sum psychopathic measures	0.01.	0.01
	(0.00)	(0.01)
positive feedback*sum psychopathic measures	−0.01.	−0.01
	(0.00)	(0.01)
negative feedback*sum psychopathic measures	−0.01	−0.00
	(0.13)	(0.01)

The interaction terms are regressed separately, controlling for gender, game version, maximise, narcissism and conditions.

Standard errors for coefficients shown in parenthesis; Significance level: ‘***’ < 0.001 ‘**’ < 0.01 ‘*’ < 0.05 and ‘.’ < 0.1.

At the same time, playing the deception/non-deception version had a slight effect on the overall cooperation, but it had a strong and statistically significant effect on CaC: participants playing the deception version of the game were more inclined to maintain cooperative behaviour compared to participants who were informed about the real identity of their opponent. However, there are no differences between low and high psychopathic players when looking at the game version (Table 8). In this sense, these results confirm our second hypotheses, proving that the opponent’s identity affects individual’s decisions.

Two other factors appeared to influence players’ cooperative behaviours: *gender* and *maximise*. As reported in the literature^25^, females show more cooperative behaviour than males, and this situation was confirmed by our experiment; in addition the effect was stronger when looking at the inclination of participants to maintain cooperative behaviour.

The maximise variable expressed the participants’ willingness to maximise their own profit during the game. As shown in the correlation matrix (Table 5), maximise is positively correlated with coldheartedness, a factor of psychopathy: the more players were goal oriented (goal = maximise their own profit), the higher their score in the subscale of psychopathy. Furthermore, though the impact was very small, we found that the more the participants aimed to achieve a high score, the less they cooperated with their partners.

Overall, our results record a main effect of fearless dominance on both overall cooperation and CaC and a main effect of the game version in the maintenance of cooperative strategies. In addition, we observed two other main effects: gender and maximise both had a negative effect on cooperation and cooperation over time.

### Strategies

The second main objective of this study was to identify players’ strategies. Here we report only the four most significant strategies (Table 9), while the complete results can be found in the online SI. The four strategies were selected to represent the main categories of strategies, given the results reported in the online SI. 2TF2T was the most frequently chosen strategy amongst the forgiving strategies, TF3T amongst the cooperative ones, and Grim amongst the unforgiving ones, while ALLD is the representative for defective strategies.

**Table 9:** Percentages of individuals adopting each one of the four strategies. Statistical significance describes how significantly different from zero are the percentages estimated through MLE. We also show the correlation matrix between the strategies adopted by each participant and their psychopathic traits.

Gamma	TF3T	2TF2T	Grim	ALLD
**Percentage of participants adopting the selected strategies**.				
0.88**	0.15	0.22	0.07	0.55**
(0.13)	(0.10)	(0.11)	(0.05)	(0.08)
**Correlation matrix between strategies adopted and psychopathic traits**.				
	**TF3T**	**2TF2T**	**Grim**	**ALLD**
Fearless Dominance	−0.03	0.01	−0.01	0.01
Self-centred impulsivity	0.14*	−0.08	0.07	−0.06
Coldheartedness	0.20**	−0.19**	0.05	0.12.

Bootstrapped standard errors (shown in parentheses) used to calculate p-values. Significance level: ‘***’ < 0.001 ‘**’ < 0.01 ‘*’ < 0.05 and ‘.’ < 0.1.

Table 9 reports the percentages of players adopting such strategies, where the statistical significance depends on how different those percentages are from zero. The estimates are obtained via maximum likelihood estimation for each subject separately, and the results reported are the average over the 192 participants of the experiment. In other words, we calculated the probability of each participant to adopt each one of the selected strategies, maximising the estimations in such a way to minimise the error individually for each subject. Gamma represents the averaged error in the estimation of such probabilities, with γ < 1 representing a good approximation.

More than half of the participants adopted a strategy that was approximated as the purely defective ALLD. This is in line with the high level of defection recorded during the experiment. The rest of the players divided between unforgiving strategies such as Grim (7%), and forgiving strategies such as TF3T and 2TF2T (37%).

Coldheartedness strongly influenced the use of both TF3T and 2TF2T, and self-centred impulsivity had also an impact on the adoption of TF3T. It is interesting to notice the contradictory correlations of coldheartedness: while higher levels of this sub-scale are positively correlated with TF3T, it is negatively correlated with 2TF2T. Both strategies are considered forgiving and nice, nevertheless, they have opposite correlations with this sub-scale of psychopathy. Furthermore, both self-centred impulsivity and coldheartedness are positively correlated with TF3T, suggesting that the higher participants were in two of the three sub-scales of psychopathy, the more they adopted such a strategy. It is interesting the absence of effect of fearless dominance on strategy selection despite its effect on overall cooperation and cooperation after cooperation. Considering such contradictory results, it would be interesting to observe a longer game, to study the effect on psychopathic traits of strategy selection in greater depth, untangling these correlations.

## Discussion

This study focuses on the interaction between personality traits and cooperation in the framework of an iterated Prisoner’s Dilemma in the presence of emotional facial feedback. Our finding of a significant negative correlation between the fearless dominance score and cooperation is surprising. Previous studies typically found significant relationships between self-centred impulsivity^4^ and cooperation, with a particular correlation between Impulsive Nonconformity and Machiavellian Egocentricity, two sub-scales that partially contribute to the self-centred impulsivity scale. Based on our findings, we suggest that the fearlessness and stress immunity sub-scales buffered the threat of retaliatory defection, urging participants to risk continued defection in pursuit of higher gains. This is supported by assumptions suggesting that high fearless dominance traits may provide an adaptive advantage to the individual^6^. An increase in boldness and a decrease in fear would allow the individual to take calculated risks to obtain greater rewards^26,27^.

Although a marginal interaction effect between the treatments and psychopathic traits was recorded, the absence of significance of the facial feedback conditions on the player’s cooperation is puzzling. A possible interpretation is that the participants may have interpreted seeing the emotional reactions of their opponent as an attempt at manipulation towards a cooperative goal, resulting in a retaliatory defection. Moreover, they could have interpreted the computer feedback as unrealistic, thus, they might not have taken that factor into consideration in the decision-making process. This is a limitation of our study and the only real solution would be to remove the computer component of the game and use a real-life opponent. Another explanation, and potential confounder, were the experiment instructions. They emphasised the game-like nature of the PD, specifically encouraging participants to earn the most points. These directions may have created an atmosphere of competition, where it is socially acceptable to maximise your own benefits, even at a cost to your opponent, instead of a social exchange scenario. This would explain why defection levels were so high and also why negative emotional feedback was not effective in deterring defection.

Our study also adds interesting findings to the literature investigating the relationship between the opponent’s identity and participants’ performance. Comparing the strategies of players in the deception version with those who were aware they were playing against a computer, we can see that dealing with a human opponent drives participants towards a more consistent cooperative behaviour.

Moreover, this study adopted an important line of inquiry which looked directly at participants’ sequence of decisions throughout the entire game, estimating which strategies were more likely to be adopted. Although the estimated coefficients describing the percentages of players adopting each specific strategy are not significantly different from zero (apart from the completely defective strategy ALLD), it is interesting to observe some significant correlations between the probability of adopting a particular strategy and the participants’ psychopathic traits. It would be interesting to investigate this point further, allowing for a longer game, as having a longer pattern of decisions will allow for a better estimation of the strategies used.

Despite the limitations encountered, our work adds complexity and insight to the body of work investigating psychopathic traits and social interactions within an affective feedback environment. Future research should take into consideration the potential of face-to-face interactions to investigate psychopathy effects on decision-making in a more realistic scenario, and a more thorough analysis of player’s strategies to identify patterns typical for specific personality traits.

## Methods

### Personality measures

The PPI-R is a 154-item self-report questionnaire^6^ on psychopathic traits with 8 sub-scales, and seven of the eight subscales can be grouped into two main factors: Fearless Dominance and Self-centred Impulsivity, while Coldheartedness is considered as an additional factor. In this study, we implemented a 40-item version of the PPI-R^28^ (see online SI for sample questions). Additionally, a recently developed method, the IRS-10, allowed us to test the response reliability of participants in the PPI-R-40^29^. Participants with IRS-10 scores above the cut-off were deemed to have completed the PPI-R-40 in an inconsistent and therefore unreliable manner and were eliminated from analysis, leading to the exclusion of 14 participants. The cut-off score was set at an IRS-10 at the 95th percentile or higher (≥13).

In addition to the PPI-R-40, participants also completed the Narcissistic Personality Inventory-16 (NPI-16)^30^. The NPI-16 is a 16-question short-form version of the original NPI-40^31^. The NPI-16 has shown a high correlation with the original NPI-40 (r = 0.90, p < 0.001) and, in addition, it has been shown to possess sufficient internal, discriminant and predictive validity.

### Data collection

A total of 233 participants were initially recruited via an online platform, Prolific Academic https://www.prolific.ac. Complete data were available for 206 participants; 14 participants were excluded due to inconsistencies in their responses^6^. The final sample was composed of 192 participants (112 female, age: *M* = *34.5, SD* = *11.6*). Participants received a small compensation of £2, independent of their achievement in the game.

### Procedure

After an introduction page explaining the structure of the game, participants were introduced to a presumed opponent, showing a picture of one of the two individuals (from DISFA). Participants were instructed to play so as to achieve the highest score possible. At the end of the 30 rounds, participants completed the PPI-R-40, and the NPI-16, followed by a feedback page which included yes/no questions such as “Did you believe you were playing against a real person?” and “Did you try to maximise your own profit?”. Participants were then debriefed about the nature of the game and the computer based opponent.

## Supplementary information

Supplementary Information

## Data Availability

The datasets generated during the current study are available in the Open Science Framework repository, https://osf.io/d5czr/?view_only=fdac94a2713a403aa9f692ac65ca6c53.
