# Supplementary material for: The effect of psychopathy on cooperative strategies in an iterated Prisoner’s Dilemma experiment with emotional feedback
Source: Sci Rep. 2019 Feb 19;9:2299. doi: 10.1038/s41598-019-38796-0 (PMC6381135; doi:10.1038/s41598-019-38796-0)
Supplement: Supplementary file 1 — Supplementary Information [file 41598_2019_38796_MOESM1_ESM.pdf]

The effect of psychopathy on cooperative strategies in an iterated Prisoner's Dilemma experiment with emotional feedback.Supplementary Information.

Martina Testori[1,*,+], **Thehela OA Harris**[2,+], **Rebecca B Hoyle**[1], **and Hedwig Eisenbarth**[3,4]

[1]University of Southampton, Department of Mathematical Sciences, Southampton, SO17 1BJ, UK
[2]University of Southampton, Department of Medicine, Southampton, SO17 1BJ, UK
[3]University of Southampton, Department of Psychology, Southampton, SO17 1BJ, UK
[4]Victoria University of Wellington, School of Psychology, Wellington, 6012, New Zealand
[*]m.testori@soton.ac.uk
[+]shared first authorship

Regression analysis

K-fold cross-validation procedure

To select the best fitting model for our dataset, a 5-fold cross-validation procedure was implemented. As stated in the main body, three regression models were considered for all three dependent variables, namely the Generalised Linear Model (GLM), the Beta-Binomial Model (BBM) and the Logistic Model (LM). The technique was implemented with the package "cvTools" in R.

GLM and BBM's performances were remarkably close for all three dependent variables, while LM showed a bad fit in all three cases, see Table A.

Table A. Repeated 5-fold cross-validation results: estimated prediction errors.

DV:	overall cooperation	cooperation after cooperation	cooperation after defection
GLM	0.196 *	0.257 *	0.234 *
BBM	0.199	0.257	0.239
LM	0.646	0.734	0.777

* selected model

Additional results

For completeness, we report the results for the variable CaD: cooperation after a previous defection. As for CaC, this is calculated as the number of times a participant cooperated immediately following a previous defection. The same analyses reported in the main manuscript were implemented on CaD. Table B reports the results of the regression analysis, while Table C reports the interaction terms. None variable under observation had a significant impact on the participants' tendency to move towards a more cooperative strategy after a previous defection. Similarly, the interaction terms between the conditions implemented in the experiment and the psychopathic traits had no effect on the individuals' tendency to move towards cooperative behaviours.

Table B. GLM results for participants' cooperation after a previous defection CaD Basic regressions without covariates and complete regressions with covariates for all three dependent variables.

DV	CaD	CaD
intercept	0.40 ***	0.49 ***
	(0.02)	(0.13)
gender		-0.03
		(0.05)
maximise		-0.01
		(0.02)
fearless dominance	-0.01	-0.01 .
	(0.00)	(0.001)
self-centred impulsivity	0.00	0.00
	(0.00)	(0.00)
coldheartedness	-0.01	-0.01
	(0.01)	(0.01)
narcissism	0.29	0.26
	(0.28)	(0.29)
positive feedback		-0.10
		(0.07)
negative feedback		-0.04
		(0.07)
positive *negative feedback		0.02
		(0.09)
game version		-0.01
		(0.05)

Standard errors for coefficients shown in parenthesis; Significance level: '***' <0.001 '**' <0.01 '*' <0.05 '.' <0.1

Table C. Interaction terms in GLM models for participants' cooperation after defection, considering participants' cumulative measure of psychopathy.

DV :	cooperation after defection (CaD)
game version*sum psychopathic measures	0.01
	(0.00)
positive*negative feedback*sum psychopathic measures	0.01
	(0.00)
positive feedback*sum psychopathic measures	-0.01
	(0.00)
negative feedback*sum psychopathic measures	-0.01
	(0.01)

The interaction terms are regressed separately, controlling for gender, game version, maximise, narcissism and conditions.
Standard errors for coefficients shown in parenthesis; Significance level: '***' <0.001 '**' <0.01 '*' <0.05 '.' <0.1

Strategy analysis

Strategies selection

In an infinite Iterated Prisoner's Dilemma there are an infinite number of strategies to choose from. For this reason, when trying to identify which strategy a participant is playing, it is important to restrict the infinite set to a finite, but still representative, sub-set of strategies.

In this analysis, we adopted the strategies selected by Fundenberg, Rand and Dreber (2012)[1]. Starting from the most known strategies analysed in the literature (Always cooperative/defective, Tit for Tat, Win-Stay,Lose-Shift), they included several variations (see Table D). These strategies capture the principal features considered in strategies: niceness, fairness, punishment, forgiveness, leniency and exploitation[1,2]. Strategies considered nice are for example ALLC, TFT, where the latter has been highly discussed to be one of the most successful strategy for the IPD. TFT and its variations are also considered to be fair strategies, giving the benefit of the doubt to the opponent before punishing him for his defective behaviour. On this point, forgiveness is another aspect often discussed when looking at strategies. T2 (formulated by Bó and Fréchette (2011)[3]) is an example of a forgiving strategy, as well as TFT and its modifications (2TFT, 2TF2T) . In contrast, Grim is considered an unforgiving strategy, as well as its lenient variations (Grim2, Grim3). Lastly, they considered those strategies which tried to exploit the opponent, by defecting most of the time (ALLD), although sometimes showing a cooperative behaviour at the beginning (CALLD). For a more detailed explanation of the strategies please see Fundenberg, Rand and Dreber (2012) online Appendix.

Furthermore, it is important to consider that completely random strategies cannot be approximated. In this sense, they included the alternating strategy, which can be seen as a random strategy, as it is not based on the opponent's decisions, but only on the individual's previous choice.

Table D. Description of the twenty strategies initially considered

Strategy	Abbreviation	Description
Always cooperate	ALLC	Cooperate in each round
Tit for tat	TFT	Cooperate until the opponent defects, then defect till the opponent cooperates again
Tit for two tat	TF2T	Cooperate until the opponent defects twice in a row, then defect till the opponent cooperates again
Tit for three tat	TF3T	Cooperate until the opponent defects three times in a row, then defect till the opponent cooperates again
Two tit for tat	2TFT	Cooperate until the opponent defects, then defect till the opponent cooperates twice in a row
Two tit for two tat	2TF2T	Cooperate until the opponent defects twice in a row, then defect till the opponent cooperates twice in a row
T2	T2	Cooperate until the opponent defects, then defects twice and return to cooperate (regardless the opponent's decisions)
Grim	Grim	Cooperate until the opponent defects, then defect forever
Lenient Grim 2	Grim2	Cooperate until the opponent defects twice in a row, then defect forever
Lenient Grim 3	Grim3	Cooperate until the opponent defect three times in a row, then defect forever
Win-Stay, Lose-Shift	WNLS	Cooperate if in the previous round both players made the same decision, defect otherwise
Win-Stay, Lose-Shift with 2 rounds of punishment	2PTFT	Cooperate if both players cooperated in the last 2 rounds, if both defected in the last two rounds or if both defected two rounds ago and cooperated in the last round. Defect otherwise
Always defect	ALLD	Defect in each round
False cooperator	C-ALLD	Cooperate in the first round then defect forever
Exploitative Tit for tat	DTFT	Defect in the first round, then play TFT
Exploitative Tit for two tat	DTF2T	Defect in the first round, then play TF2T
Exploitative Tit for three tat	DTF3T	Defect in the first round, then play TF3T
Exploitative Grim2	DGrim2	Defect in the first round, then play Grim2
Exploitative Grim3	DGrim3	Defect in the first round, then play Grim3
Alternator	DC-Alt	Defect in the first round, then alternate cooperation and defection

Maximum Likelihood Estimation for participants' strategies

As stated in the main manuscript, the procedure follows the one presented in Bó and Fréchette (2011)[3]. Supposing that each participant starts with a fixed strategy, this technique allows for errors in the pattern of decisions over the rounds. The likelihood of adopting a specific strategy s^k is calculated by allowing a deviation between the decision expected by s^k and the actual decision taken by the player at each round. Knowing the history of each participant, it is possible to deduce the next move for each of the selected strategies and calculate the difference between that decision and the one made by the participant. In a more formal way, this is translated with a function:

$$1\{s_{ir}(s^k)+\gamma\varepsilon_{ir}\geq 0\} = \begin{cases} 1 \text{ (cooperate)} & \text{if } s_{ir}(s^k)+\gamma\varepsilon_{ir}\geq 0 \\ 0 \text{ (defect)} & \text{if } s_{ir}(s^k)+\gamma\varepsilon_{ir}< 0 \end{cases} \tag{1}$$

where $1\{\cdot\}$ is an indicator function (meaning it can only take 0 and 1 as values), ir stands for subject i and round r; s^k is a specific strategy k, $s_{ir}(s^k)$ is the action implied by the strategy s^k given the history recorded (1=cooperation, -1=defection)[1], ε is the error term and γ is the variance of the error. The error term is independent across subjects, rounds, interactions and histories, and γ can be interpreted as the probability of making a mistake, supposing the strategy decision is the correct one. Moreover, the density of the error is assumed to be such that the likelihood that, over all rounds, subject i uses strategy s^k is:

$$p_i(s^k) = \prod_{R\in\mathbb{R}} \left(\frac{1}{1+exp(-s_{ir}(s^k)/\gamma)}\right)^{y_{ir}} \left(\frac{1}{1+exp(s_{ir}(s^k)/\gamma)}\right)^{1-y_{ir}}, \tag{2}$$

where y_{ir} is player's i decision at round r.

Hence, the probability of an error in the implementation of a strategy is equal to $\frac{1}{1+exp(\frac{1}{\gamma})}$. Thus, for example, if player i cooperates in round r and the expected decision, according to s^k, is to defect (-1), the internal term of Equation (2) would be equal to $\frac{1}{1+exp(\frac{1}{\gamma})}$ which tends to 0 as $\gamma\to 0$. On the contrary, if the expected decision for the strategy is cooperation, the internal term of Equation (2) would be equal to $\frac{1}{1+exp(\frac{-1}{\gamma})}$ which tends to 1 as $\gamma\to 0$.

Considering now the set of strategies $K=\{s^1,\dots,s^k\}$ under analysis, and the complete dataset collected, the log-likelihood function for the entire sample is:

$$\mathscr{L} = \sum_I ln\left(\sum_K p(s^k)p_i(s^k)\right). \tag{3}$$

Here, $p(s^k)$ represents the proportion of data which is attributed to strategy s^k. More properly, p stands for the distribution of the strategies over the dataset. If we had an infinite population, p would express the exact fraction of individuals playing s^k. As in our experiment we have a finite number of subjects, we would have a certain variance, different from zero, in the population shares.

The next step is to implement Maximum Likelihood Estimation (MLE) to estimate the γ parameter and the fractions $\{p(s^1),\dots,p(s^k)\}$ of individuals adopting the strategies we hypothesised. The MLE process is implemented in MATLAB using the *fmincon* function which is a non-linear programming solver. The function calculates the values of γ and $p(s^i)$, where $i\in\{1,\dots,k\}$, and maximises the log likelihood function, under the constraint that they belong to $[0,1]$.

The final step is to generate the standard errors for the estimated frequencies by constructing 100 bootstrap samples for the complete dataset, and then performing the MLE for γ and $p(s^i)$ on the bootstrapped samples. The standard errors are calculated by taking the standard deviation of the estimates calculated, and the t-test p-values are generated using the *normcdf* function.

Complete results

Table E reports the complete results for the 20 strategies considered. The results include both the estimation of the strategies across the population and the correlation between the strategies and the personality traits of the participants. The results reported for the percentages of participants adopting each one of the selected strategies are calculated as the mean over each participants.

[1]The different codification for cooperation and defection is based on equation (2), as one of the two members has to be equal to 1 at each round R. Any codification that satisfies that condition could be used.

We run the estimation for each player individually and we then reported the average percentages for each of the strategies. In the same way, the error Gamma is the average of the errors calculated for each player.

Table E. Percentages of individuals adopting each one of the twenty strategies. Statistical significance describes how significantly different from zero are the percentages estimated through MLE. We also show the correlation matrix between the strategies adopted by each participant and their psychopathic traits.

Percentage of participants adopting the selected strategies.										
ALLC	TFT	TF2T	TF3T	2TFT	2TF2T	Grim	Grim2	Grim3	T2	
0.009<br>(0.016)	0.010<br>(0.027)	0.002<br>(0.045)	0.099<br>(0.054)	0.032<br>(0.054)	0.111<br>(0.084)	0.037<br>(0.028)	0.027<br>(0.030)	0.002<br>(0.019)	0.038<br>(0.027)	
WSLS	2PTFT	ALLD	CALLD	DTFT	DTF2T	DTF3T	DGrim2	DGrim3	DC-alt	Gamma
0.006<br>(0.007)	0.000<br>(0.005)	0.471**<br>(0.074)	0.025<br>(0.026)	0.076<br>(0.036)	0.042<br>(0.052)	0.000<br>(0.020)	0.000<br>(0.004)	0.001<br>(0.015)	0.012<br>(0.018)	0.823 ***<br>(0.100)

Correlation matrix between strategies adopted and psychopathic traits.										
	ALLC	TFT	TF2T	TF3T	2TFT	2TF2T	Grim	Grim2	Grim3	T2
Fearless Dominance	0.028	0.019	-0.055	-0.040	0.017	0.037	0.002	0.028	- 0.041	-0.075
Self-centred implusivity	0.054	-0.092	0.085	0.174*	0.121.	-0.116	0.148*	-0.160*	0.031	-0.041
Coldheartedness	0.052	-0.120.	-0.033	0.174*	-0.031	-0.137 .	0.074	0.080	-0.006	0.049
	WSLS	2PTFT	ALLD	CALLD	DTFT	DTF2T	DTF3T	DGrim2	DGrim3	DC-alt
Fearless Dominance	0.057	-0.008	0.020	-0.023	-0.018	-0.034	-0.056	-0.013	0.020	-0.013
Self-centred implusivity	0.002	-0.023	-0.044	-0.011	0.054	-0.015	0.099	-0.023	0.039	-0.132.
Coldheartedness	-0.008	0.042	0.105	-0.031	0.055	-0.128.	-0.035	0.032	0.0126	-0.134.

Bootstrapped standard errors (shown in parentheses) used to calculate p-values.
*** Significant at the 1 percent level. ** Significant at the 5 percent level. * Significant at the 10 percent level.

Instructions and screenshots

Instructions

4% completed

Welcome!

Thank you for agreeing to participate in our "Commercial decision-making game!"

You are about to play an online game version of the Prisoner's Dilemma. In this scenario, you own an electronics store that sells various electronic goods. You will be playing against an opponent, who is one of 20 student volunteer participants. Each of these student participants have been equipped with a web-cam, so you will be able to see who you are playing against, however we do not require you to use your own web-cam. In the game, your opponent has opened another electronics store nearby to you. Since you both sell the same products, you must now decide how you wish to price your items. You have two options:

• **Standard Pricing**- where you leave the normal price of the item the same.

• **Sale Pricing**- where by lowering the price of the item, you seek to attract more customers to your store and less to your competitor, thereby making more money.

Both you and your opponent will be presented with an item for sale in the shop and you and your opponent will decide whether to give Standard or Sale pricing to the item. The in-game rewards are given depending on the choice that both you and your opponent make. There are 4 different outcomes, with the first listed option being your decision:

• Standard Pricing-Standard Pricing- **30%** profit

• Sale Pricing- Sale Pricing- **20%** profit

• Sale Pricing- Standard Pricing- **40%** profit

• Standard Pricing- Sale Pricing- **10%** profit

Points are awarded based on profit, with higher profit outcomes receiving more points. In this game, you are encouraged to **maximise you own profit** to achieve the most points at the end of the game.

At the end of each round, your opponent will have the opportunity to provide facial feedback with the use of a mute webcam from their position. There are 40 rounds to the game and at the end the points gained in each round will be tallied on both sides and the winner will be shown. Due to our use of student volunteers as game opponents, this study will only be available for completion between the hours of **8am and 10pm.**

If you are ready to begin, press "Next".

Good luck!

Next

Figure 1. Deception version

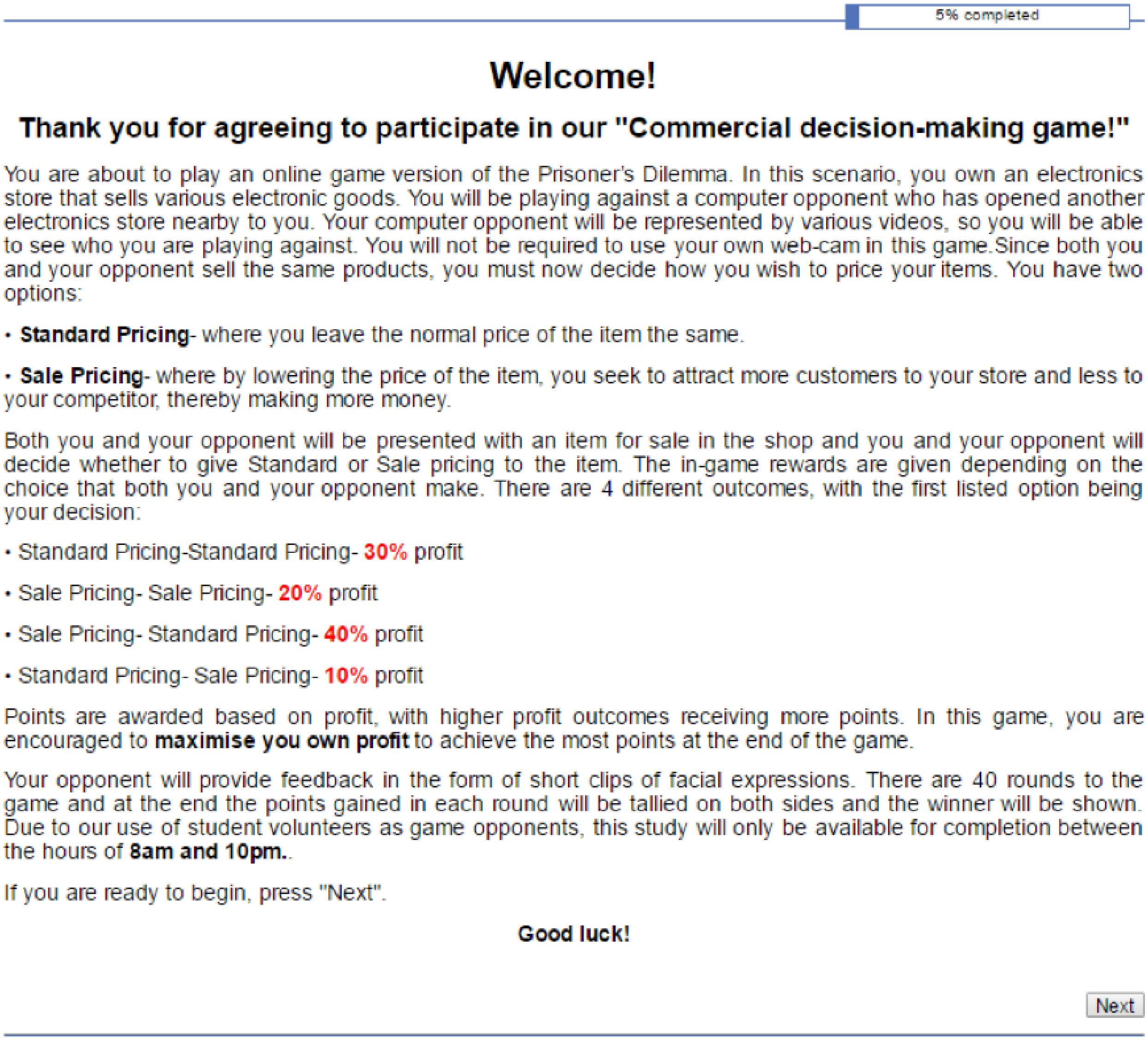

5% completed

Welcome!

Thank you for agreeing to participate in our "Commercial decision-making game!"

You are about to play an online game version of the Prisoner's Dilemma. In this scenario, you own an electronics store that sells various electronic goods. You will be playing against a computer opponent who has opened another electronics store nearby to you. Your computer opponent will be represented by various videos, so you will be able to see who you are playing against. You will not be required to use your own web-cam in this game.Since both you and your opponent sell the same products, you must now decide how you wish to price your items. You have two options:

• **Standard Pricing**- where you leave the normal price of the item the same.

• **Sale Pricing**- where by lowering the price of the item, you seek to attract more customers to your store and less to your competitor, thereby making more money.

Both you and your opponent will be presented with an item for sale in the shop and you and your opponent will decide whether to give Standard or Sale pricing to the item. The in-game rewards are given depending on the choice that both you and your opponent make. There are 4 different outcomes, with the first listed option being your decision:

• Standard Pricing-Standard Pricing- **30%** profit

• Sale Pricing- Sale Pricing- **20%** profit

• Sale Pricing- Standard Pricing- **40%** profit

• Standard Pricing- Sale Pricing- **10%** profit

Points are awarded based on profit, with higher profit outcomes receiving more points. In this game, you are encouraged to **maximise you own profit** to achieve the most points at the end of the game.

Your opponent will provide feedback in the form of short clips of facial expressions. There are 40 rounds to the game and at the end the points gained in each round will be tallied on both sides and the winner will be shown. Due to our use of student volunteers as game opponents, this study will only be available for completion between the hours of **8am and 10pm.**.

If you are ready to begin, press "Next".

Good luck!

Next

Figure 2. Non-deception version

Experiment scenario and decision page

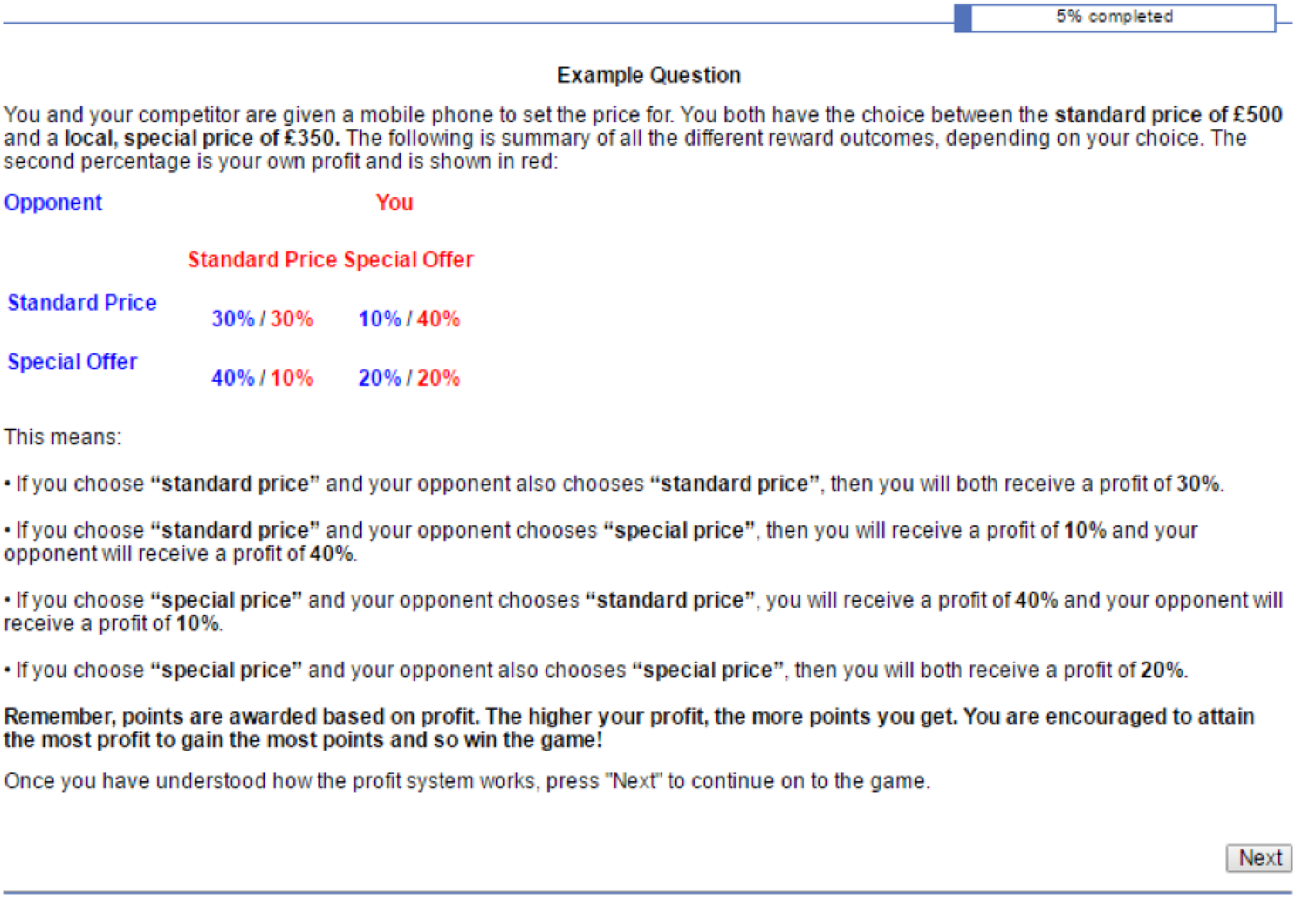

Figure 3. Experiment scenario

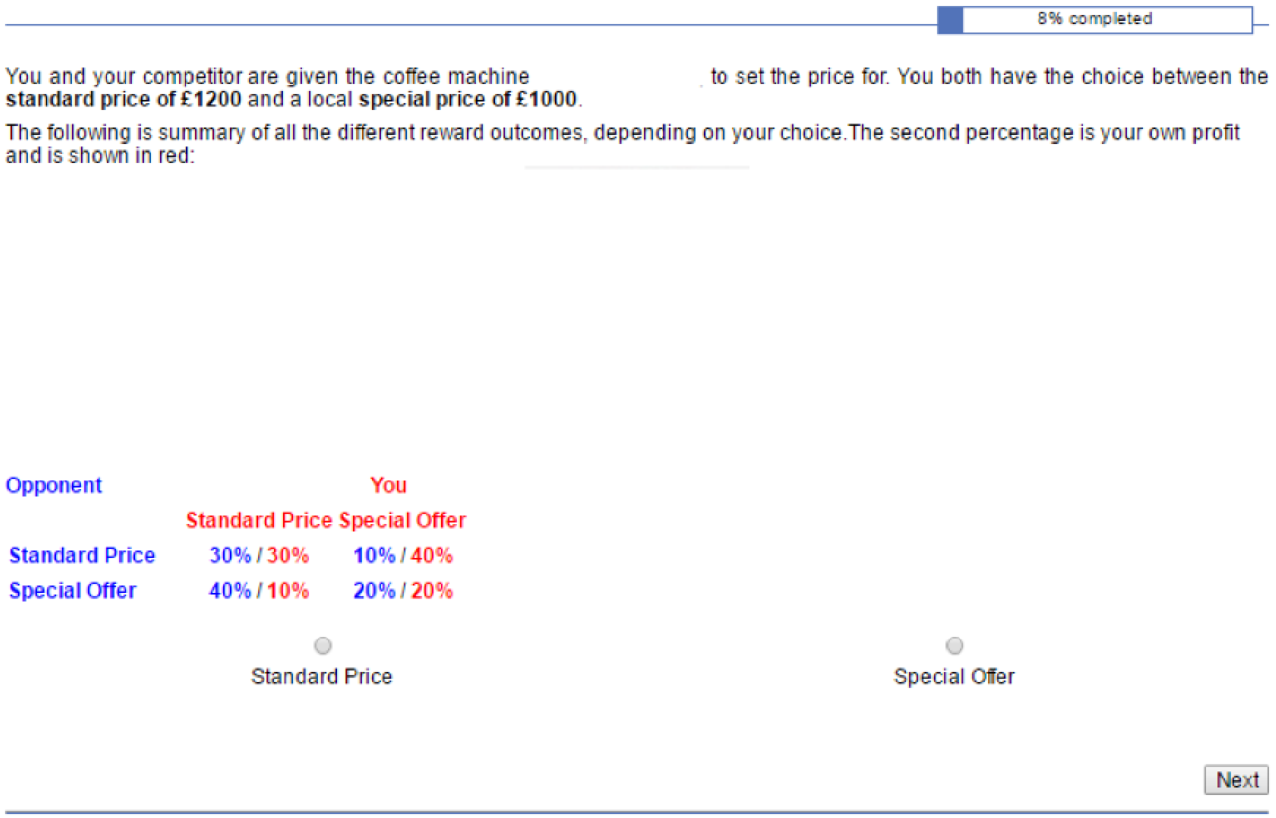

Figure 4. Decision page

Personality questionnaire

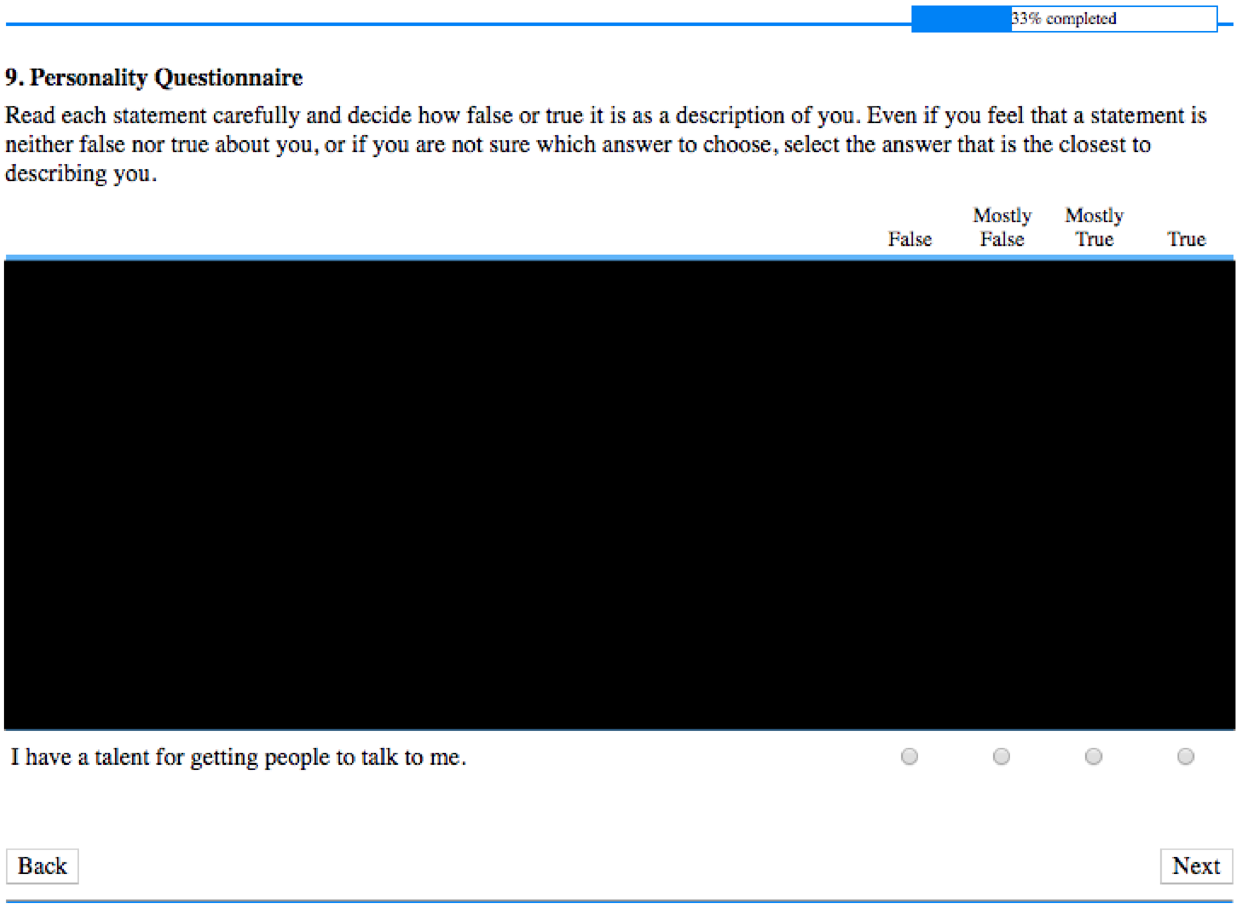

Figure 5. Personality test-sample 1

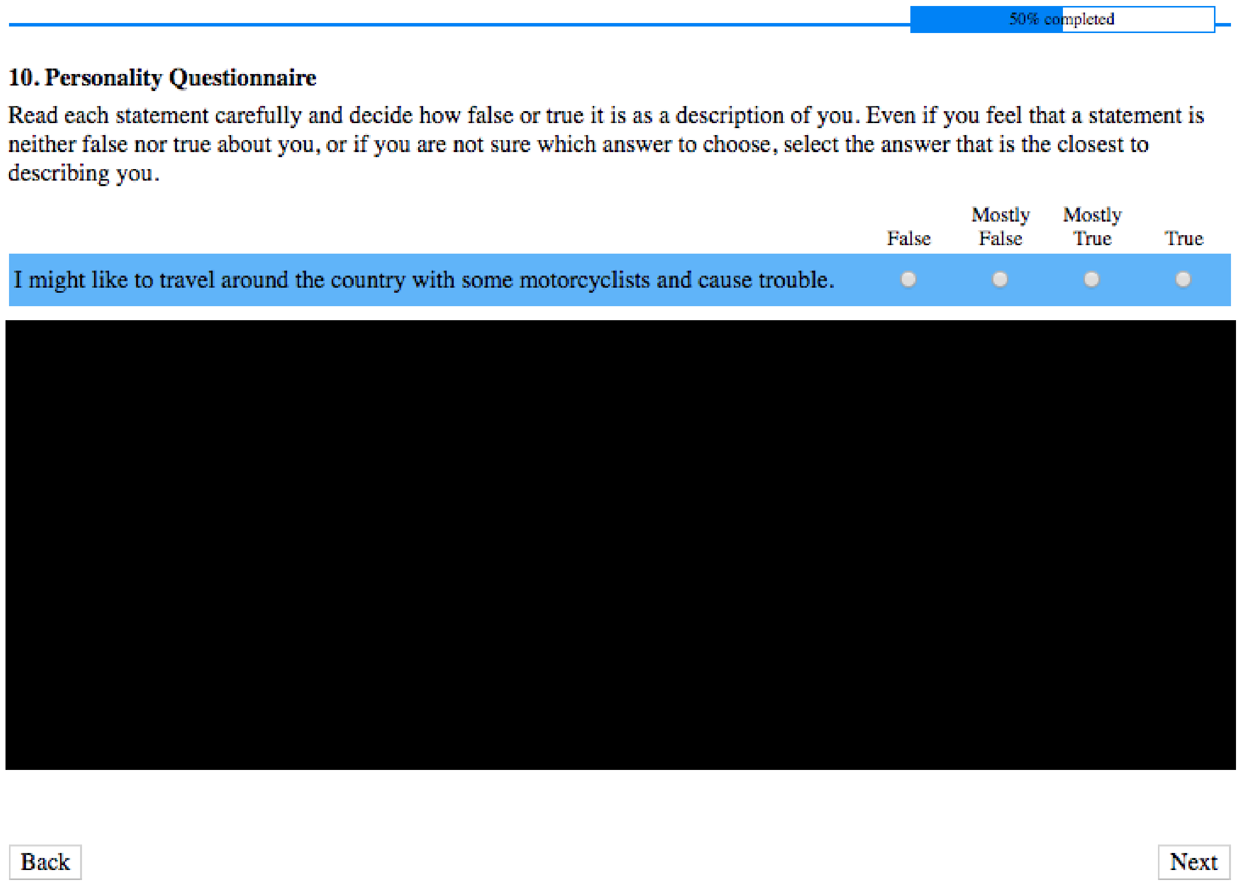

Figure 6. Personality test-sample 2

Follow-up questionnaire

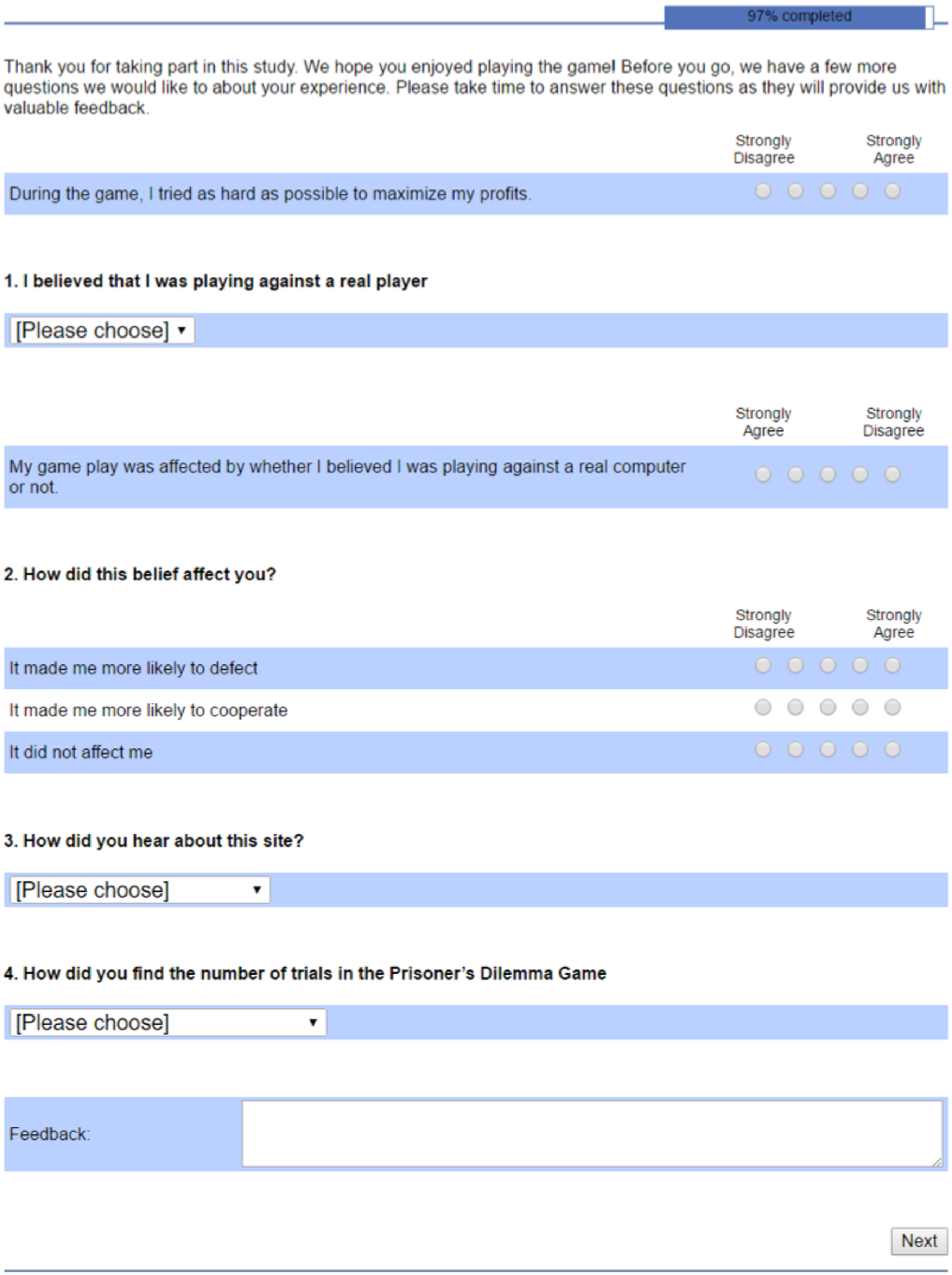
97% completed

Thank you for taking part in this study. We hope you enjoyed playing the game! Before you go, we have a few more questions we would like to about your experience. Please take time to answer these questions as they will provide us with valuable feedback.

	Strongly Disagree				Strongly Agree
During the game, I tried as hard as possible to maximize my profits.	○	○	○	○	○

1. I believed that I was playing against a real player

[Please choose] ▾

	Strongly Agree				Strongly Disagree
My game play was affected by whether I believed I was playing against a real computer or not.	○	○	○	○	○

2. How did this belief affect you?

	Strongly Disagree				Strongly Agree
It made me more likely to defect	○	○	○	○	○
It made me more likely to cooperate	○	○	○	○	○
It did not affect me	○	○	○	○	○

3. How did you hear about this site?

[Please choose] ▾

4. How did you find the number of trials in the Prisoner's Dilemma Game

[Please choose] ▾

Feedback:

Next

Figure 7. Follow-up questionnaire

References

1. Fudenberg, D., Rand, D. G. & Dreber, A. Slow to anger and fast to forgive: Cooperation in an uncertain world. *Am. Econ. Rev.* **102**, 720–49 (2012).

2. Axelrod, R. & Hamilton, W. D. The evolution of cooperation. *Science* **211**, 1390–1396 (1981).

3. Dal Bó, P. & Fréchette, G. R. The evolution of cooperation in infinitely repeated games. *Am. Econ. Rev.* **101**, 411–429 (2011).
